# Association between Resistin Levels and All-Cause and Cardiovascular Mortality: A New Study and a Systematic Review and Meta-Analysis

**DOI:** 10.1371/journal.pone.0120419

**Published:** 2015-03-20

**Authors:** Andrea Fontana, Sara Spadaro, Massimiliano Copetti, Belinda Spoto, Lucia Salvemini, Patrizia Pizzini, Lucia Frittitta, Francesca Mallamaci, Fabio Pellegrini, Vincenzo Trischitta, Claudia Menzaghi

**Affiliations:** 1 Unit of Biostatistics, IRCCS “Casa Sollievo della Sofferenza”, San Giovanni Rotondo, Italy; 2 Endocrine Unit, Department of Clinical and Molecular Biomedicine, University of Catania, Catania, Italy; 3 Scuola Superiore di Catania, University of Catania, Catania, Italy; 4 CNR-IBIM and Nephrology, Dialysis and Transplantation Unit of Reggio Calabria, Reggio Calabria, Italy; 5 Research Unit of Diabetes and Endocrine Diseases, IRCCS “Casa Sollievo della Sofferenza”, San Giovanni Rotondo, Italy; 6 Department of Experimental Medicine, Sapienza University of Rome, Rome, Italy; National University of Singapore, SINGAPORE

## Abstract

**Context:**

Studies concerning the association between circulating resistin and mortality risk have reported, so far, conflicting results.

**Objective:**

To investigate the association between resistin and both all-cause and cardiovascular (CV) mortality risk by 1) analyzing data from the Gargano Heart Study (GHS) prospective design (n=359 patients; 81 and 58 all-cause and CV deaths, respectively); 2) performing meta-analyses of all published studies addressing the above mentioned associations.

**Data Source and Study Selection:**

MEDLINE and Web of Science search of studies reporting hazard ratios (HR) of circulating resistin for all-cause or CV mortality.

**Data Extraction:**

Performed independently by two investigators, using a standardized data extraction sheet.

**Data Synthesis:**

In GHS, adjusted HRs per one standard deviation (SD) increment in resistin concentration were 1.28 (95% CI: 1.07-1.54) and 1.32 (95% CI: 1.06-1.64) for all-cause and CV mortality, respectively. The meta-analyses included 7 studies (n=4016; 961 events) for all-cause mortality and 6 studies (n=4,187: 412 events) for CV mortality. Pooled HRs per one SD increment in resistin levels were 1.21 (95% CI: 1.03-1.42, Q-test p for heterogeneity<0.001) and 1.05 (95% CI: 1.01-1.10, Q-test p for heterogeneity=0.199) for all-cause and CV mortality, respectively. At meta-regression analyses, study mean age explained 9.9% of all-cause mortality studies heterogeneity. After adjusting for age, HR for all-cause mortality was 1.24 (95% CI: 1.06-1.45).

**Conclusions:**

Our results provide evidence for an association between circulating resistin and mortality risk among high-risk patients as are those with diabetes and coronary artery disease.

## Introduction

Several studies indicate that resistin, a 12.5 kDa cysteine-rich pro-inflammatory adipokine, is a major promoter of atherosclerosis and related cardiovascular (CV) disease [1–7], including heart failure [8].

Since CV disease is the first cause of mortality worldwide, resistin becomes a natural candidate to investigate as a possible mortality risk factor. In fact, conflicting results have been reported on this subject [9–16].

To deeper understand the role of resistin on mortality rate, we firstly added new evidences by trying to replicate our previous finding [14] in a second, independent sample of diabetic patients followed over time for both all-cause and CV mortality and then performed a meta-analysis of all published prospective studies on the association between circulating resistin and mortality risk of either all-cause or CV origin.

## Materials and Methods

### Study Population

#### GHS-prospective design

This study comprises 368 patients with type 2 diabetes mellitus (T2DM) (ADA 2003 criteria) and coronary artery disease (CAD) who were consecutively recruited at the Endocrine Unit of IRCCS “Casa Sollievo della Sofferenza” in San Giovanni Rotondo (Gargano, Center East Coast of Italy) from 2001 to 2008, as recently described [14, 17]. All patients had either a stenosis >50% in at least one coronary major vessel at coronary angiography or a previous myocardial infarction (MI). Follow-up information on outcomes was collected yearly from 2002 to 2011. The only exclusion criterion was the presence of poor life expectancy for non diabetes-related diseases. The end-point was CV mortality and all-cause mortality. Confirmation of the event was obtained from death certificates (i.e. according to the international classification of diseases’ codes: 428.1- ninth edition—and I21.0-I21.9, I25.9, I46.9-I50.9, I63.0, I63.9, I70.2—tenth edition).

Clinical data were obtained from a standardized interview and examination. Smoking habits and history of hypertension, dyslipidemia and MI as well as glucose-lowering treatment were also recorded at time of examination. Data regarding medications were confirmed by review of medical records.

Serum resistin was measured in 359 (98%) participants by a commercial ELISA (Bio Vendor, Brno Czech Republic) at the Research Unit of Diabetes and Endocrine Diseases in San Giovanni Rotondo, as previously described [18].

### Ethics

The study was approved by the Institutional Ethic Committee IRCCS “Casa Sollievo della Sofferenza”, San Giovanni Rotondo. All participants gave written consent.

### Meta-Analysis of Prospective Studies

#### Search strategy and selection criteria

The present meta-analysis follows the Preferred Reporting Items for Systematic Review and Meta-analyses (PRISMA) statements (S1 PRISMA Checklist). Two investigators (S.S. and C.M.) independently searched MEDLINE and Web of Science for prospective studies with resistin as exposure and CV or all-cause mortality as outcome, published until October 2014. Search terms used were (Resistin[Mesh] or RETN) and (Mortality [Mesh] or death).

Reference lists of retrieved articles (online S1 Supporting Information) were hand-searched for additional studies. Data gathered included: first author, publication year, location, study design, race/ethnicity, number of participants, number of events, proportion of males, duration of follow-up, age, mean resistin levels and body mass index, events ascertainment and variables controlled for. Each article identified by this search process was considered if it was a cohort study published as original (abstracts, letters, reviews, and meta-analyses had been excluded) and reported the adjusted hazard ratios (HR) or the natural logarithm of the HR (logHR) estimates, along with their 95% confidence interval (95%CI) or standard error (SE) or p-value, for each study-specific non-reference exposure level. When unavailable, the reported unadjusted HRs were considered. Moreover, studies where HRs were expressed in terms of unitary increment of their resistin standard deviation (SD) were also considered.

#### Data preparation

Preparation of the data of the original studies for the meta-analysis followed two stages. First, since different studies used different units of measure to express serum resistin concentration doses, all the measures were converted into ng/ml as the standard measure.

Second, as the levels of serum resistin concentration were often reported as the minimum-maximum range for each tertile or quartile of the study-specific resistin distribution, the representative dose value was assigned for each dose group by fitting a gamma distribution to the serum resistin distribution over the range’s class. To estimate gamma parameters, study-specific resistin mean and SD were used as suggested in Hartemink et al. [19]. When resistin mean and SD were unavailable for a specific-study, median and interquartile range were used instead, respectively. For the open-ended risk classes (e.g., <2.7 or > 4.9), the representative value was assigned following the algorithms suggested by Il’yasova et al. [20].

### Statistics

Patients’ baseline characteristics were reported as mean ± SD and percentages for continuous and categorical variables, respectively.

The time variable was defined as the time between the baseline examination and date of the event, or, for subjects who did not experience the event, the date of the last available clinical follow-up. Incidence rates for all-cause and CV mortality were expressed as the number of events per total number of person-years (py). Risks were reported as HRs along with 95% CI.

Dose-response meta-analysis followed a two-step process and was performed for all-cause and CV mortalities outcomes, separately.

Step 1: We considered those studies in which HRs were reported with respect to a study-specific categorical reference dose of serum resistin concentrations (i.e. tertiles or quartiles) [9, 11–13]: study-specific slopes of logHR across exposure categories were estimated with weighted log-linear models (WLM), using study-specific rescaled doses (with respect to the reference representative dose) as covariate and variances of logHRs (derived from SE or 95%CI or p-values reported within each study) as weights. No intercept term was included in the model since, by definition, when the rescaled dose is zero the linear function would start from logHR = 0. Moreover, to account for between logHRs correlations within each study, plausible pairwise covariance values were derived on the basis of known logHRs variances and possible correlations values: 0, 0.2, 0.3, 0.5, 0.8, 1. Covariance values for which the model achieved the minimum Akaike’s Information Criterion (AIC) were chosen.

Step 2: We considered those studies in which HRs were reported with respect to one increment of resistin SD (scaled unit of measure) [10, 14–16] and the current study: study-specific slopes (logHRs) were estimated.

Step 3: Given that resistin measurement was carried out in different specimens (i.e. serum or plasma), by different laboratories using different assays, we firstly converted the scaled measures with respect to one increment of study-specific SD, and then all study-specific slopes obtained from Step 1 and Step 2 were combined and their summary statistics were estimated. Statistical heterogeneity among studies was assessed using the Cochran Q-test and heterogeneity hold for p-values less than 0.10 [21]. Study-specific estimates were pooled using either the fixed-effects model or, in presence of heterogeneity (i.e. Q-test statistically significant), the random effects model [22]. Furthermore, to explain residual heterogeneity, meta-regression analyses were performed using those study-level covariates which were available from all studies, including age, sex, smoking habits, follow-up time, BMI, presence of diabetes, hypertension and specimen of resistin measurement.

To evaluate consistency and stability of HRs estimates found from meta-analysis and meta-regression, sensitivity analyses were carried out, by re-running all analyses after excluding one study at one time.

Due to the small number of studies (fewer than ten) and the presence of substantial between-study heterogeneity, following Sterne et al. [23] and Ioannidis et al. [24] recommendations, we did not perform tests for publication bias.

Forest plots were shown, where a square was plotted for each study whose center projection on the underlying scale corresponded to the study-specific HR. The area of the square was proportional to the inverse of the variance of the logHR and thus gives a measure of the amount of statistical information available from that particular estimate. A diamond was used to plot the summary HRs, the center of which represents the HR; the extremes of the summary HRs show the 95% CI.

Bubble plot of study-specific logHRs is shown against the chosen study-level covariate which explained the most proportion of heterogeneity. The size of each bubble was inversely proportional to the standard error of specific logHR.

Two-sided p-values<0.05 were considered for statistical significance. Statistical analysis were performed using SAS Software, Release 9.3 (SAS Institute, Cary, NC, USA) and R 2.15 (package: metafor).

## Results

### The GHS-prospective design

Clinical features of study participants are summarized in Table 1.

**Table 1:** Clinical characteristics of patients from GHS-prospective design (n = 359).

Sex (% males)	67.4
Age (yrs)	64.5±8.1
Smokers (%)	45
Diabetes duration (yrs)	13.8±9.2
BMI (kg/m^2^)	30.2±4.8
HbA_1C_ (%)	8.6±1.9
Total cholesterol (mg/dL)	175.8±45.7
HDL-cholesterol (mg/dL)	43.6±14.6
non-HDL-cholesterol (mg/dL)	131.3±43.5
LDL-cholesterol (mg/dL)	100.9±38.6
Triglycerides (mg/dL)	152.6±91.8
Glucose-lowering therapy	
Diet only (%)	7
Oral agents (%)	35
Insulin ± oral agents (%)	58
Antihypertensive therapy (%)	85
Antidyslipidemic therapy (%)	65
hsCRP (mg/L)	6.1±12.7
Resistin (ng/ml)	10.7±6.6

Continuous variables were reported as mean± standard deviation whereas categorical variables as percentages.

GHS: Gargano Heart Study; BMI: body mass index; HbA1c: glycated haemoglobin; HDL: high density lipoprotein; LDL: low density lipoprotein; hsCRP: high sensitivity C-reactive protein.

During follow-up (5.4 ±2.5 years for a total of 1,934 patient-years, py), 81 all-cause and 58 CV deaths occurred, corresponding to an annual incidence rate of 4.2 and 3.0 events per 100 person-years, respectively.

Each SD (i.e. 6.6 ng/ml) increment of serum resistin was associated with an increased risk of both all-cause and CV mortality (HR = 1.33, 95%CI: 1.17–1.52, p = 2x10^-5^ and HR = 1.34, 95%CI: 1.14–1.57, p = 3x10^-4^, respectively; Table 2).

**Table 2:** Risk of all-cause and cardiovascular mortality per one standard deviation (i.e. 6.6 ng/ml) increment of serum resistin levels in the GHS-prospective design.

	Model 1		Model 2	
Outcome	HR (95% CI)	p value	HR (95% CI)	p value
All-cause mortality	1.33 (1.17–1.52)	2x10^-5^	1.28 (1.07–1.54)	0.008
Cardiovascular mortality	1.34 (1.14–1.57)	3x10^-4^	1.32 (1.06–1.64)	0.013

GHS, Gargano Heart Study.

Model 1: unadjusted

Model 2: adjusted for age, sex, smoking habit, BMI, HbA1c, hsCRP, anti-diabetic, anti-hypertensive andanti-dyslipidemic treatments.

These associations were independent of clinical features including age, sex, smoking habits, BMI, HbA1c, hsCRP, anti-diabetic, anti-hypertensive and anti-dyslipidemic treatments (Table 2). Similar results were obtained after adding into the model also LDL and non HDL (p = 0.014 and p = 0.018, respectively) cholesterol or when hsCRP was replaced by fibrinogen into the model (p = 0.03 and p = 0.05, respectively).

### Meta-Analysis of Prospective Studies

**Fig 1:**
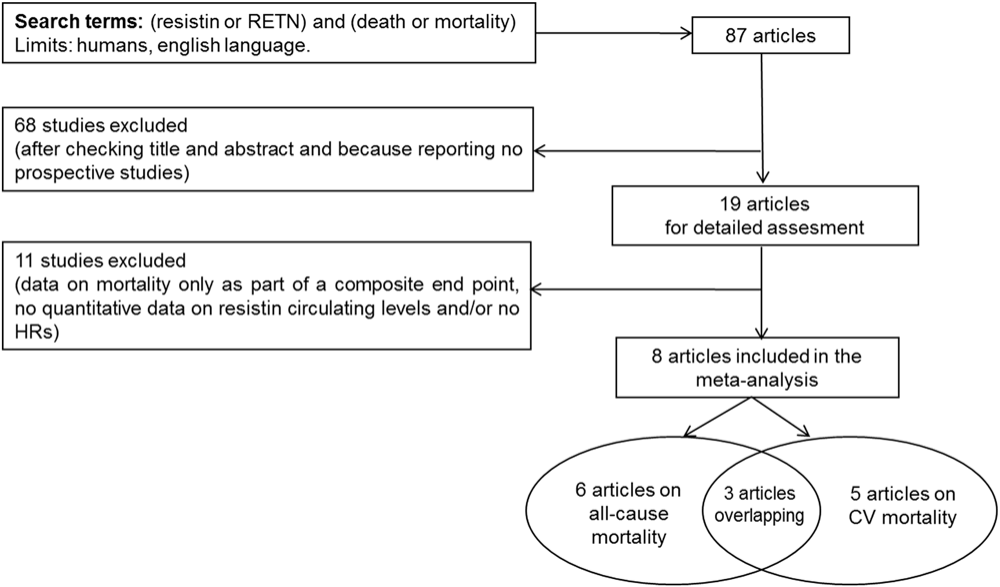
Search strategy for selecting studies to include in meta-analyses of resistin and all-cause mortality and cardiovascular mortality (search last run on October 2014).

Eighty-seven hits from MEDLINE and Web of Science were retrieved (S1 Supporting Information). After the exclusion of all articles which did not meet the inclusion criteria (Fig. 1 and S1 Supporting Information), 6 independent studies on all-cause mortality [9, 11–14, 16] and 5 independent studies on CV mortality [10–12, 15, 16] were eligible for inclusion.

One hundred and three participants from our previous study on all-cause mortality, overlapped with those of the current study [14]. For this reason we have included, in the meta-analysis for all-cause mortality, HR already reported in Menzaghi et al. [14] after the exclusion of the 103 overlapping patients. In all, with the inclusion of the present GHS, data from a total of 4,016 (961 incident cases) and 4,187 (412 incident cases) participants were meta-analyzed for either all-cause or CV mortality respectively. Main features of all these studies are shown in Tables 3 and 4.

**Table 3:** Main features of prospective studies included in the meta-analyses.

First author, (ref)	Ethnicity	Clinical set	Subjects (n)	Cause of mortality	Mean F-U (yrs)	Resistin (ng/ml)	Deaths (n)
Pilz et al. [11]	European	General population (78% w/ CAD)	1162	All-cause/CV	5.5	3.6 (2.7–5.0)*	198
Efstathiou et al. [9]	European	IS	211	All-cause	5.0	26.1±9.7*	101
Lubos et al. [10]	European	IS	1888	CV	2.6	5 (4.0–6.7)§	70
Lee et al. [12]	Korean	MI	397	All-cause/CV	1.0	19.52 (14.3–25.5)* ^	28
Zhang et al. [13]	American	CHD	980	All-cause	6.1	8.5 (5.8–12.2)§	274
Menzaghi et al. [14]	European	T2DM	676	All-cause	7.8	10.1±8.3§	114
Spoto et al. [16]	European	ESRD	231	All-cause/CV	4.8	127.2±23.3*	165
Silva et al. [15]	European	T2DM	150	CV	3.0	6.1±3.5§	35
Current study	European	T2DM (100% w/ CAD)	359	All-cause/CV	5.4	10.7±6.6§	81

F-U: follow-up; CAD: coronary artery disease; IS: ischemic stroke; MI: myocardial infarction; CHD: coronary heart disease; T2DM: type 2 diabetes mellitus; ESRD: end stage renal disease.

When not differently indicated, data are reported as mean ± standard deviation (SD).

^Approximated mean from median and approximated SD from interquartile range (IQR): under the assumption of normal distribution, then IQR ≈ 1.35*SD.

*Data are plasma resistin concentration.

§ Data are serum resistin concentration.

**Table 4:** Clinical measurements available in all prospective studies included in the meta-analyses.

First author, (ref)	Age (years)*	BMI (Kg/m^2^)*	Smokers (%)	Males (%)	Diabetes (%)	Hypertension (%)
Pilz et al. [11]	64.1±11.3^	26.7±3.6^	62.5	69.2	28.9	54.7
Efstathiou et al. [9]	69.1±13.8	27.5±4.7	33.0	57.3	28.4	70.1
Lubos et al. [10]	61.1±9.8	27.8±3.9	20.0	79.1	83.1	74.5
Lee et al. [12]	62.3±11.3	24.0±3.4	29.3	72	28.3	51.0
Zhang et al. [13]	66.5±10.8	28.5±5.3	19.8	81.3	26.8	70.5
Menzaghi et al. [14]	61.5±9.7	31.0±5.7	20.7	47.7	100	46.2
Spoto et al. [16]	60.0±15.0	24.5±4.4	37.2	55	15.2	36.0
Silva et al. [15]	62.7±11.0	26.3±2.7	N.A.	61.3	100	N.A.
Current study	64.5±8.1	30.2±4.8	44.5	67.4	100	85.0

When not differently indicated, data are reported as mean ± standard deviation (SD).

^Approximated mean from median and approximated SD from interquartile range (IQR): under the assumption of normal distribution, then IQR ≈ 1.35*SD.

All studies were conducted in high risk patients with previous CAD [11, 13], MI [12], ischemic stroke [9, 10], T2DM [14, 15] or end stage renal disease [16] (Table 3). With the exception of Lee et al. [12] and Zhang et al. [13], studies were carried out in Europeans (Table 3), with a very similar study mean age, but a quite different study mean BMI (Table 4).

#### All-cause mortality (n = 7 studies)

The number of subjects from each study ranged from 211 to 1162 with mean follow-up ranging from 1.0 to 7.8 years (Table 3).

Four studies [9, 11, 12, 14] reported a significant association as we have shown in our current study. One displayed a directionally consistent but statistically insignificant association [13], and the last one showed no association at all [16].

**Fig 2:**
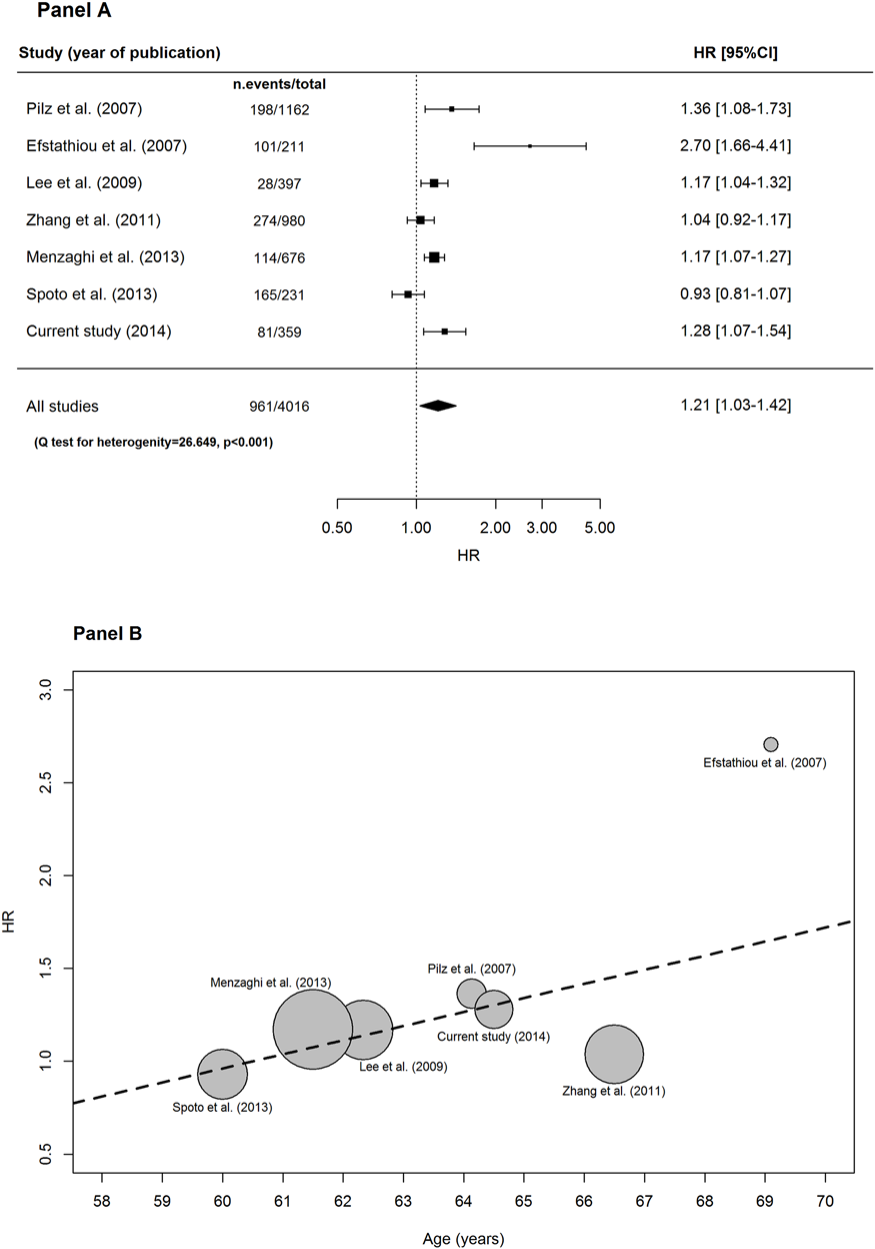
Forest plot for random-effects meta-analysis (panel A) and bubble plot for random-effects meta-regression with age as study-level covariate (panel B) on all-cause mortality.

Fig. 2, panel A, shows the random effects meta-analysis for all-cause mortality whose pooled HR per one increment in SD of resistin concentration was 1.21 (95%CI:1.03–1.42, p = 0.020).

A significant heterogeneity across studies was indeed observed (Q-test p for heterogeneity <0.001). Sensitivity and meta-regression analyses were then performed (see Statistical Methods section). At sensitivity analysis, HR estimates per each unitary increment of SD in resistin levels turned out to be stable (ranging from HR = 1.13 to HR = 1.26) and quite consistent with the HR obtained from all studies. At meta-regression analysis, among all study-level covariates which were available from all studies (Table 4), study mean age was the only one which was significantly associated with all-cause mortality (HR = 1.06, 95%CI:1.00–1.13, p = 0.049) (Fig. 2, panel B), explaining 9.9% of the observed heterogeneity. The pooled HR per one SD resistin increment, estimated from a meta-regression including study mean age was equal to 1.24 (95%CI:1.06–1.45, p = 0.006).

#### CV mortality (n = 6 studies)

The number of subjects from each study ranged from 150 to 1888 with a mean follow-up ranging from 1.0 to 5.5 years (Table 3).

With the only exception of our current study, none of the previous ones reported a significant association with CV mortality [10–12, 15, 16], though 3 studies displayed a directionally consistent association [10–12].

**Fig 3:**
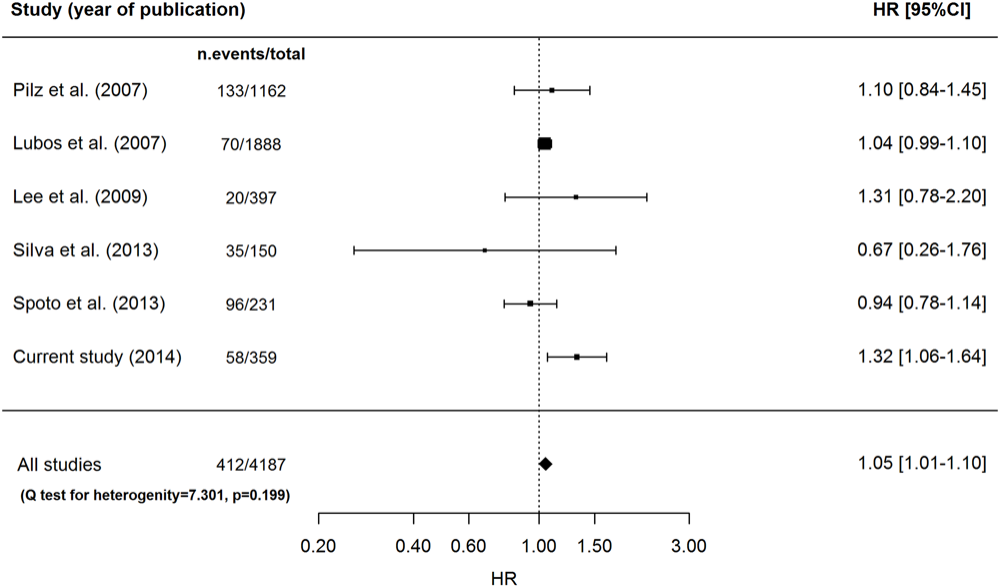
Forest plot for random-effects meta-analysis on CV mortality.

The pooled HR per one increment in SD of resistin concentration for CV mortality was 1.05 (95%CI: 1.01–1.10; p = 0.016), using the fixed-effects model (Fig. 3).

No significant heterogeneity across study results was observed (Q-test p for heterogeneity = 0.20), so that no meta-regression analysis was carried out.

## Discussion

Data obtained from the GHS-prospective design confirm our previous observation that serum resistin is a predictor of all-cause mortality [14] and extends the role of resistin also to CV mortality. In addition, the role of resistin on both all-cause and CV mortality is further confirmed by our meta-analyses carried out in all eligible published studies, most of which described, in fact, some association [9, 11, 12, 14]. Although these positive results have to be taken with caution, investigating the association also with various non-fatal CVD events would help to deeper understand the role of resistin on atherosclerosis [25].

Since heterogeneity was observed across studies for all-cause mortality, meta-regression analysis was employed to get more reliable pooled HRs estimates. Although the observed results have to be taken with great caution because of the limited number of available studies, heterogeneity was partly explained by study mean age (i.e. stronger association in studies with older subjects).

Though our meta-regression allows speculate that age acts as a modulator of the resistin effect on all-cause mortality, only studies with individual patient data may say a last word about this hypothesis. Unfortunately, no studies addressing this specific issue are available.

Finally, we observed a potential risk for publication bias; however, given the small number of studies (fewer than ten) and the presence of between-study heterogeneity, we believed unnecessary to address it in accordance with previous recommendations [23, 24].

We have to recognize some study limitations, as follows. Our meta-analyses have been conducted in high-risk patients (mostly diabetic and/or with CV disease or with end stage renal disease); so, it is unknown whether our finding applies also to “healthy” individuals with low risk profile. In addition, most studies included in the meta-analyses were carried out in patients from European Countries, thus making impossible to generalize our findings to population of different ancestry and/or environmental background.

In conclusion, our results provide evidence for an association between higher circulating resistin levels and increased mortality risk. Further studied are needed to verify whether or not including resistin in risk engines which are already validated [26–28] improves our ability to predict the risk of mortality, especially in high-risk individuals.

## Supporting Information

S1 PRISMA Checklist (PDF)

S1 Supporting Information List of the full-text articles retrieved from MEDELINE and Web of Science. (DOCX)
