# Supplementary material for: Association between Resistin Levels and All-Cause and Cardiovascular Mortality: A New Study and a Systematic Review and Meta-Analysis
Source: PLoS One. 2015 Mar 20;10(3):e0120419. doi: 10.1371/journal.pone.0120419 (PMC4368155; doi:10.1371/journal.pone.0120419)
Supplement: S1 Supporting Information — (DOCX) [file pone.0120419.s002.docx]

S1 Supporting information

List of the full-text articles retrieved from MEDELINE and Web of Science.

[1-87]

1. Beauloye V, Zech F, Tran HT, Clapuyt P, Maes M, Brichard SM. Determinants of early atherosclerosis in obese children and adolescents. J Clin Endocrinol Metab 2007;92: 3025-3032.
2. Bobbert P, Jenke A, Bobbert T, Kuhl U, Rauch U, Lassner D, et al. High leptin and resistin expression in chronic heart failure: adverse outcome in patients with dilated and inflammatory cardiomyopathy. European Journal of Heart Failure 2012;14: 1265-1275.
3. Boehncke S, Fichtlscherer S, Salgo R, Garbaraviciene J, Beschmann H, Diehl S, et al. Systemic therapy of plaque-type psoriasis ameliorates endothelial cell function: results of a prospective longitudinal pilot trial. Arch Dermatol Res 2011;303: 381-388.
4. Boehncke S, Salgo R, Garbaraviciene J, Beschmann H, Ackermann H, Boehncke WH, et al. Changes in the sex hormone profile of male patients with moderate-to-severe plaque-type psoriasis under systemic therapy: results of a prospective longitudinal pilot study. Arch Dermatol Res 2011;303: 417-424.
5. Boehncke S, Salgo R, Garbaraviciene J, Beschmann H, Hardt K, Diehl S, et al. Effective continuous systemic therapy of severe plaque-type psoriasis is accompanied by amelioration of biomarkers of cardiovascular risk: results of a prospective longitudinal observational study. J Eur Acad Dermatol Venereol 2011;25: 1187-1193.
6. Chang JH, Jung JY, Lee HH, Chung W, Joo KW, Kim S. Serum Resistin as a Novel Marker of Erythropoietin Resistance in Nondiabetic Patients on Hemodialysis. Tohoku Journal of Experimental Medicine 2011;224: 281-285.
7. Cheng JM, Akkerhuis KM, Battes LC, van Vark LC, Hillege HL, Paulus WJ, et al. Biomarkers of heart failure with normal ejection fraction: a systematic review. Eur J Heart Fail 2013;15:1350-1362.
8. Chung W, Jung ES, Shin D, Choi SH, Jung JY, Chang JH, et al. Low Resistin Level is Associated with Poor Hospitalization-Free Survival in Hemodialysis Patients. Journal of Korean Medical Science 2012;27:377-381.
9. Daniel P, Lesniowski B, Jasinska A, Pietruczuk M, Malecka-Panas E. Usefulness of Assessing Circulating Levels of Resistin, Ghrelin, and IL-18 in Alcoholic Acute Pancreatitis. Digestive Diseases and Sciences 2010;55: 2982-2987.
10. da Silva TF, Levy-Neto M, Bonfa E, Pereira RMR. High Prevalence of Metabolic Syndrome in Takayasu Arteritis: Increased Cardiovascular Risk and Lower Adiponectin Serum Levels. Journal of Rheumatology 2013;40: 1897-1904.
11. de Leon AC, Perez MDR, Gonzalez DA, Diaz BB, Coello SD, Hernandez AG, et al. Hemodynamics and Metabolism at Low versus Moderate Altitudes. High Altitude Medicine & Biology 2011;12: 179-186.
12. Doh FM, Chang TI, Koo HM, Lee MJ, Shin DH, Kim CH, et al. The effect of HMG-CoA reductase inhibitor on insulin resistance in patients undergoing peritoneal dialysis. Cardiovasc Drugs Ther 2012;26: 501-509.
13. Dong XQ, Hu YY, Yu WH, Zhang ZY. High concentrations of resistin in the peripheral blood of patients with acute basal ganglia hemorrhage are associated with poor outcome. Journal of Critical Care 2010;25: 243-247.
14. Dong XQ, Yang SB, Zhu FL, Lv QW, Zhang GH, Huang HB. Resistin is associated with mortality in patients with traumatic brain injury. Critical Care 2010;14: R190.
15. Duffy SL, Lagrone L, Herndon DN, Mileski WJ. Resistin and Postburn Insulin Dysfunction. Journal of Trauma-Injury Infection and Critical Care 2009;66: 250-254.
16. Efstathiou SP, Tsiakou AG, Tsioulos DI, Panagiotou TN, Pefanis AV, Achimastos AD, et al. Prognostic significance of plasma resistin levels in patients with atherothrombotic ischemic stroke. Clinica Chimica Acta 2007;378: 78-85.
17. Erer HB, Sayar N, Guvenc TS, Aksaray S, Yilmaz H, Altay S, et al. Prognostic value of serum resistin levels in patients with acute myocardial infarction. Kardiologia Polska 2014;72: 181-186.
18. Fang WQ, Zhang Q, Peng YB, Chen M, Lin XP, Wu JH, et al. Resistin level is positively correlated with thrombotic complications in Southern Chinese metabolic syndrome patients. Journal of Endocrinological Investigation 2011;34: E36-E42.
19. Filippidis G, Liakopoulos V, Mertens PR, Kiropoulos T, Stakias N, Verikouki C, et al. Resistin serum levels are increased but not correlated with insulin resistance in chronic hemodialysis patients. Blood Purification 2005;23: 421-428.
20. Gherman C, Mironiuc A, Palcau L, Cristea A, Muresan A, Filip A, et al. Adipocytokines and their relationship with symptomatic atherosclerotic peripheral arterial disease. Revista Romana De Medicina De Laborator 2010;18: 23-32.
21. Golledge J, Clancy P, Jamrozik K, Norman PE. Obesity, adipokines, and abdominal aortic aneurysm - Health in men study. Circulation 2007;116: 2275-2279.
22. Gouni-Berthold I, Berthold HK, Chamberland JP, Krone W, Mantzoros CS. Short-term treatment with ezetimibe, simvastatin or their combination does not alter circulating adiponectin, resistin or leptin levels in healthy men. Clin Endocrinol (Oxf) 2008;68: 536-541.
23. Gonzalez-Gay MA, Garcia-Unzueta MT, Berja A, Gonzalez-Juanatey C, Miranda-Filloy JA, Vazquez-Rodriguez TR, et al. Anti-TNF-alpha therapy does not modulate leptin in patients with severe rheumatoid arthritis. Clinical and Experimental Rheumatology 2009;27: 222-228.
24. Gonzalez-Gay MA, Vazquez-Rodriguez TR, Garcia-Unzueta MT, Berja A, Miranda-Filloy JA, de Matias JM, et al. Visfatin is not associated with inflammation or metabolic syndrome in patients with severe rheumatoid arthritis undergoing anti-TNF-alpha therapy. Clinical and Experimental Rheumatology 2010;28: 56-62.
25. Gruzdeva O, Uchasova E, Belik E, Dyleva Y, Shurygina E, Barbarash O. Lipid, adipokine and ghrelin levels in myocardial infarction patients with insulin resistance. BMC Cardiovasc Disord. 2014; 14: 7
26. Ho YL, Lin YH, Lee CM, Hsu RB, Ting HT, Chou NK, et al. Prognostic significance of adipocytokines and extracellular matrix activity in heart failure patients with high B-type natriuretic peptide. Clinical Biochemistry 2009;42: 1407-1412.
27. Hoffmann MM, Pilz S, Weihrauch G, Seelhorst U, Wellnitz B, Winkelmann BR, et al. Effect of the resistin-420C > G polymorphism on cardiovascular disease and mortality. Clinical Endocrinology 2008;69: 344-345.
28. Hu W, Liu CW, Su J, Lu J, Zhu Y, Liu BW. Elevated plasma visfatin concentrations in patients with community-acquired pneumonia. Peptides 2013;43 :8-12.
29. Kang Y, Park HJ, Kang MI, Lee HS, Lee SW, Lee SK, et al. Adipokines, inflammation, insulin resistance, and carotid atherosclerosis in patients with rheumatoid arthritis. Arthritis Research & Therapy 2013;15: R194
30. Kaplan JM, Denenberg A, Monaco M, Nowell M, Wong H, Zingarelli B. Changes in peroxisome proliferator-activated receptor-gamma activity in children with septic shock. Intensive Care Medicine 2010;36: 123-130.
31. Karbowska A, Boratynska M, Klinger M. Resistin: A pathogenic factor or a biomarker of metabolic disorders and inflammation? Postepy Higieny I Medycyny Doswiadczalnej 2009;63: 485-491.
32. Kataoka H, Sharma K. Renal handling of adipokines. Obesity and the Kidney 2006;151: 91-105.
33. Kaynar K, Kural BV, Ulusoy S, Cansiz M, Akcan B, Misir N, et al. Is there any interaction of resistin and adiponectin levels with protein-energy wasting among patients with chronic kidney disease. Hemodialysis International 2014;18: 153-162.
34. Khan RS, Kato TS, Chokshi A, Chew M, Yu SQ, Wu C, et al. Adipose Tissue Inflammation and Adiponectin Resistance in Patients With Advanced Heart Failure Correction After Ventricular Assist Device Implantation. Circulation-Heart Failure 2012;5: 340-348.
35. Kim JY, Choi EY, Mun HS, Min PK, Yoon YW, Lee BK, et al. Usefulness of metabolic syndrome score in the prediction of angiographic coronary artery disease severity according to the presence of diabetes mellitus: relation with inflammatory markers and adipokines. Cardiovascular Diabetology 2013;12: 140.
36. Koch A, Gressner OA, Sanson E, Tacke F, Trautwein C. Serum resistin levels in critically ill patients are associated with inflammation, organ dysfunction and metabolism and may predict survival of non-septic patients. Critical Care 2009;13: R95.
37. Krecki R, Krzeminska-Pakula M, Drozdz J, Szczesniak P, Peruga JZ, Lipiec P, et al. Relationship of serum angiogenin, adiponectin and resistin levels with biochemical risk factors and the angiographic severity of three-vessel coronary disease. Cardiology Journal 2010;17: 599-606.
38. Krecki R, Krzeminska-Pakula M, Peruga JZ, Szczesniak P, Lipiec P, Orszulak-Michalak D, et al. Influence of treatment strategy on serum adiponectin, resistin and angiogenin concentrations in patients with stable multivessel coronary artery disease after one-year follow-up. Kardiologia Polska 2010;68: 1313-1322.
39. Krecki R, Krzeminska-Pakula M, Peruga JZ, Szczesniak P, Lipiec P, Wierzbowska-Drabik K, et al. Elevated resistin opposed to adiponectin or angiogenin plasma levels as a strong, independent predictive factor for the occurrence of major adverse cardiac and cerebrovascular events in patients with stable multivessel coronary artery disease over 1-year follow-up. Medical Science Monitor 2011;17: CR26-CR32.
40. Lederer DJ, Kawut SM, Wickersham N, Winterbottom C, Bhorade S, Palmer SM, et al. Obesity and Primary Graft Dysfunction after Lung Transplantation The Lung Transplant Outcomes Group Obesity Study. American Journal of Respiratory and Critical Care Medicine 2011;184: 1055-1061.
41. Lee CT, Ng HY, Hsu CY, Tsai YC, Yang YK, Chen TC, et al. Proinflammatory Cytokines, Hepatocyte Growth Factor and Adipokines in Peritoneal Dialysis Patients. Artificial Organs 2010;34: E222-E229.
42. Lee SH, Ha JW, Kim JS, Choi EY, Park S, Kang SM, et al. Plasma adiponectin and resistin levels as predictors of mortality in patients with acute myocardial infarction: data from infarction prognosis study registry. Coronary Artery Disease 2009;20: 33-39.
43. Li Y, Zhang LH, Gu Y, Hao CM, Zhu TY. insulin resistance as a predictor of cardiovascular disease in patients on peritoneal dialysis. Peritoneal Dialysis International 2013;33: 411-418.
44. Liangos O, Domhan S, Schwager C, Zeier M, Huber PE, Addabbo F, et al. Whole Blood Transcriptomics in Cardiac Surgery Identifies a Gene Regulatory Network Connecting Ischemia Reperfusion with Systemic Inflammation. Plos One 2010;5: e13658.
45. Longenecker CT, Dunn W, Jiang Y, Debanne SM, McComsey GA. Adipokines and vascular health in treated HIV infection: an obesity paradox? Aids 2013;27:1353-6.
46. Lubos E, Messow CM, Schnabel R, Rupprecht HJ, Espinola-Klein C, Bickel C, et al. Resistin, acute coronary syndrome and prognosis results from the AtheroGene study. Atherosclerosis 2007;193: 121-128.
47. Malyszko J, Kozminski P, Malyszko J, Mysliwiec M. Type of arteriovenous fistula, NYHA class and apelin in hemodialyzed patients. International Urology and Nephrology 2011;43: 185-190.
48. Malyszko J, Malyszko JS, Pawlak K, Wolczynski S, Mysliwiec M. Apelin, a Novel Adipocytokine, in Relation to Endothelial Function and Inflammation in Kidney Allograft Recipients. Transplantation Proceedings 2008;40: 3466-9.
49. Menzaghi C, Bacci S, Salvemini L, Mendonca C, Palladino G, Fontana A, et al. Serum Resistin, Cardiovascular Disease and All-Cause Mortality in Patients with Type 2 Diabetes. Plos One 2013;8: e64729.
50. Niemczyk S, Romejko-Ciepielewska K, Niemczyk L. Adipocytokines and sex hormone disorders in patients with chronic renal failure (CRF). Endokrynologia Polska 2012;63:148-155.
51. O'Donovan A, Neylan TC, Metzler T, Cohen BE. Lifetime exposure to traumatic psychological stress is associated with elevated inflammation in the Heart and Soul Study. Brain Behavior and Immunity 2012;26: 642-649.
52. Ortega-Deballon P, Duvillard L, Scherrer ML, Deguelte-Lardiere S, Bourredjem A, Petit JM, et al. Preoperative adipocytokines as a predictor of surgical infection after colorectal surgery: a prospective survey. International Journal of Colorectal Disease 2014;29: 23-29.
53. Owens CD, Kim JM, Hevelone ND, Hamdan A, Raffetto JD, Creager MA, et al. Novel adipokines, high molecular weight adiponectin and resistin, are associated with outcomes following lower extremity revascularization with autogenous vein. Journal of Vascular Surgery 2010;51: 1152-1159.
54. Pathak A, Galinier M, Senard JM. Cardiac disease of the obese. Sang Thrombose Vaisseaux 2007;19: 93-104.
55. Pilz S, Weihrauch G, Seelhorst U, Wellnitz B, Winkelmann BR, Boehm BO, et al. Implications of resistin plasma levels in subjects undergoing coronary angiography - The Ludwigshafen Risk and Cardiovascular health (LURIC) Study. Clinical Endocrinology 2007;66: 380-386.
56. Prugger C, Luc G, Haas B, Arveiler D, Machez E, Ferrieres J, et al. Adipocytokines and the risk of ischemic stroke: The PRIME Study. Annals of Neurology 2012;71: 478-486.
57. Prugger C, Luc G, Haas B, Morange PE, Ferrieres J, Amouyel P, et al. Multiple Biomarkers for the Prediction of Ischemic Stroke The PRIME Study. Arteriosclerosis Thrombosis and Vascular Biology 2013;33: 659-366.
58. Rathmann W, Herder C. Adiponectin and cardiovascular mortality: Evidence for "Reverse epidemiology". Hormone and Metabolic Research 2007;39:1-2.
59. Rho YH, Chung CP, Solus JF, Raggi P, Oeser A, Gebretsadik T, et al. Adipocytokines, Insulin Resistance, and Coronary Atherosclerosis in Rheumatoid Arthritis. Arthritis and Rheumatism 2010;62: 1259-1264.
60. Rienstra M, Sun JX, Lubitz SA, Frankel DS, Vasan RS, Levy D, et al. Plasma resistin, adiponectin, and risk of incident atrial fibrillation: the Framingham Offspring Study. Am Heart J 2012;163: 119-124.e1.
61. Salman S, Uzum AK, Telci A, Alagol F, Ozbey NC. Serum adipokines and low density lipoprotein subfraction profile in hypopituitary patients with growth hormone deficiency. Pituitary 2012;15: 386-392.
62. Santoro S, Milleo FQ, Malzoni CE, Klajner S, Borges PCM, Santo MA, et al. Enterohormonal changes after digestive adaptation: Five-year results of a surgical proposal to treat obesity and associated diseases. Obesity Surgery 2008;18:17-26.
63. Santos M, Reis A, Goncalves F, Ferreira-Pinto MJ, Cabral S, Torres S, et al. Adiponectin Levels Are Elevated in Patients With Pulmonary Arterial Hypertension. Clinical Cardiology 2014;37: 21-25.
64. Schernthaner GH, Schernthaner G. Insulin resistance and inflammation in the early phase of type 2 diabetes: Potential for therapeutic intervention. Scandinavian Journal of Clinical & Laboratory Investigation 2005;65: 30-40.
65. Schutte AE, Huisman HW, Van Rooyen JM, Schutte R, Malan L, Reimann M, et al. Should obesity be blamed for the high prevalence rates of hypertension in black South African women? Journal of Human Hypertension 2008;22: 528-536.
66. Shen LJ, Yang SB, Lv QW, Zhang GH, Zhou J, Guo M, et al. High plasma adiponectin levels in patients with severe traumatic brain injury. Clinica Chimica Acta 2014;427: 37-41.
67. Shirai K. Obesity as the core of the metabolic syndrome and the management of coronary heart disease. Current Medical Research and Opinion 2004;20: 295-304.
68. Silva AP, Fragoso A, Silva C, Viegas C, Tavares N, Guilherme P, et al. What Is the Role of Apelin regarding Cardiovascular Risk and Progression of Renal Disease in Type 2 Diabetic Patients with Diabetic Nephropathy? Biomed Research International 2013;2013: 247649.
69. Sjowall C, Cardell K, Bostrom EA, Bokarewa MI, Enocsson H, Ekstedt M, et al. High prevalence of autoantibodies to C-reactive protein in patients with chronic hepatitis C infection: association with liver fibrosis and portal inflammation. Human Immunology 2012;73: 382-388.
70. Spoto B, Mattace-Raso F, Sijbrands E, Pizzini P, Cutrupi S, D'Arrigo G, et al. Resistin and all-cause and cardiovascular mortality: effect modification by adiponectin in end-stage kidney disease patients. Nephrology Dialysis Transplantation 2013;28: 181-187.
71. Stewart PA, Luks J, Roycik MD, Sang QXA, Zhang JF. Differentially Expressed Transcripts and Dysregulated Signaling Pathways and Networks in African American Breast Cancer. Plos One 2013;8 : e82460.
72. Swindell WR, Cummings SR, Sanders JL, Caserotti P, Rosano C, Satterfield S, et al. Data Mining Identifies Digit Symbol Substitution Test Score and Serum Cystatin C as Dominant Predictors of Mortality in Older Men and Women. Rejuvenation Research 2012;15:405-13.
73. Valassi E, Biller BMK, Klibanski A, Misra M. Adipokines and Cardiovascular Risk in Cushing's Syndrome. Neuroendocrinology 2012;95: 187-206.
74. Vassiliadi DA, Tzanela M, Kotanidou A, Orfanos SE, Nikitas N, Armaganidis A, et al. Serial changes in adiponectin and resistin in critically ill patients with sepsis: Associations with sepsis phase, severity, and circulating cytokine levels. Journal of Critical Care 2012;27: 400-409.
75. Wade CE, Mora AG, Shields BA, Pidcoke HF, Baer LA, Chung KK, et al. Signals from fat after injury: Plasma adipokines and ghrelin concentrations in the severely burned. Cytokine 2013;61: 78-83.
76. Windham BG, Griswold ME, Farasat SM, Ling SM, Carlson O, Egan JM, et al. Influence of Leptin, Adiponectin, and Resistin on the Association Between Abdominal Adiposity and Arterial Stiffness. American Journal of Hypertension 2010;23: 501-507.
77. Wu XM, Lin YH, Chen A, Hsu TP, Wu YW, Lin HJ, et al. Prognostic significance of adipocytokines in systolic heart failure patients. European Journal of Clinical Investigation 2012;42: 1079-1086.
78. Yagmur E, Trautwein C, Gressner AM, Tacke F. Resistin serum levels are associated with insulin resistance, disease severity, clinical complications, and prognosis in patients with chronic liver diseases. American Journal of Gastroenterology 2006;101: 1244-1252.
79. Yildirim M, Erkan ME, Asik M, Ucgun T, Yilmaz A, Ilce HT, et al. Resistin predicts ischemia in myocardial perfusion scintigraphy. Turkish Journal of Medical Sciences 2014;44: 496-500.
80. Yin WH, Wei J, Huang WP, Chen JW, Young MS, Lin SJ. Prognostic value of circulating adipokine levels and expressions of adipokines in the myocardium of patients with chronic heart failure. Circ J 2012;76: 2139-2147.
81. Youn JC, Kim C, Park S, Lee SH, Kang SM, Choi D, et al. Adiponectin and progression of arterial stiffness in hypertensive patients. International Journal of Cardiology 2013;163: 316-319.
82. Yuan H, Weng CY, Yang YB, Huang LH, Xing XW. Resistin, an adipokine, may affect the improvement of insulin sensitivity in the metabolic syndrome patient treated with metformin. Medical Hypotheses 2013;81: 969-971.
83. Zhang MH, Na B, Schiller NB, Whooley MA. Association of Resistin With Heart Failure and Mortality in Patients With Stable Coronary Heart Disease: Data From the Heart and Soul Study. Journal of Cardiac Failure 2011;17: 24-30.
84. Zheng H, Xu HF, Xie NZ, Huang JL, Fang H, Luo M. Association of serum resistin with peripheral arterial disease. Polskie Archiwum Medycyny Wewnetrznej-Polish Archives of Internal Medicine 2013;123: 680-685.
85. Wacharasint P, Boyd JH, Russell JA, Walley KR. One size does not fit all in severe infection: obesity alters outcome, susceptibility, treatment, and inflammatory response. Critical Care 2013;17: R122.
86. Kitatani N, Taniguchi A. Association of serum resistin with TNF system activity in Japanese type 2 diabetic patients. Diabetes & Metabolism 2007;33: 156-157.
87. Balducci S, Zanuso S, Nicolucci A, Fernando F, Cavallo S, Cardelli P, et al. Anti-inflammatory effect of exercise training in subjects with type 2 diabetes and the metabolic syndrome is dependent on exercise modalities and independent of weight toss. Nutrition Metabolism and Cardiovascular Diseases 2010;20: 608-617.

List of the full-text articles excluded from meta-analyses.

Reasons:

- Articles excluded based on title and abstract:

[1,3-7,9,12,18,22-24,27,28,31,32,35,43,44,50-54,56,58,60,62,64,67,71,73,81,82,85-87]

- Articles excluded because reporting no prospective studies:

[2,10,11,15,19-21,25,29,30,33,34,37,40,41,45,47,48,57,59,61,63,65,66,69,74-76,78,79,84]

- Articles excluded because reporting no quantitative data on resistin circulating levels and/or no hazard ratios:

[13,14,26,36,72,77]

- Articles excluded because data of mortality are presented as part of a composite endpoint:

[8,17,38,39,80]

Final articles included in the meta-analyses

[16,42,46,49,55,68,70,83]

Raw data on resistin levels and association with related variables from GHS-prospective design can be provided upon request for collaborative purposes.
